# Intrinsic Localized Modes in Proteins

**DOI:** 10.1038/srep18128

**Published:** 2015-12-11

**Authors:** Adrien Nicolaï, Patrice Delarue, Patrick Senet

**Affiliations:** 1 Department of Physics, Applied Physics and Astronomy, Rensselaer Polytechnic Institute, Troy, 12180 New York, United States; 2 Laboratoire Interdisciplinaire Carnot de Bourgogne, UMR 6303 CNRS-Univ. Bourgogne Franche-Comté, 9 Av. A. Savary, BP 47 870, F-21078 Dijon Cedex, France

## Abstract

Protein dynamics is essential for proteins to function. Here we predicted the existence of rare, large nonlinear excitations, termed intrinsic localized modes (ILMs), of the main chain of proteins based on all-atom molecular dynamics simulations of two fast-folder proteins and of a rigid *α*/*β* protein at 300 K and at 380 K in solution. These nonlinear excitations arise from the anharmonicity of the protein dynamics. The ILMs were detected by computing the Shannon entropy of the protein main-chain fluctuations. In the non-native state (significantly explored at 380 K), the probability of their excitation was increased by a factor between 9 and 28 for the fast-folder proteins and by a factor 2 for the rigid protein. This enhancement in the non-native state was due to glycine, as demonstrated by simulations in which glycine was mutated to alanine. These ILMs might play a functional role in the flexible regions of proteins and in proteins in a non-native state (i.e. misfolded or unfolded states).

Intrinsic localized modes (ILMs)^1^, are members of the large soliton family^2^. They have been predicted and observed in crystals and anti-ferromagnetic materials^3^,^4^ and arise from the anharmonicity of interatomic potentials and from the discreteness of matter at the atomic scale. We expect proteins in solution (water) to subtend ILMs, that is, strongly *localized* waves, because of their well-known anharmonicity^5^,^6^,^7^,^8^,^9^,^10^,^11^. Despite the importance of protein dynamics for biological function^12^,^13^,^14^,^15^,^16^, the actual occurence of ILMs in proteins remains to be demonstrated both theoretically and experimentally.

Referring to the anharmonicity of hydrogen bonds between the amide N-H bonds and the carbonyl C = O groups of the protein backbone, Davydov proposed the existence of localized waves in *α*-helices several decades ago^17^. Using a one-dimensional quantum Hamiltonian, Davydov predicted the self-localization of C = O bond vibrations through their anharmonic coupling with the low-frequency modes of the polypeptide chain^17^. According to his proposal, this localized wave would provide a mechanism to propagate energy within a protein^18^. Experimental evidence of Davydov’s solitons in proteins continues to remain elusive^19^,^20^,^21^,^22^. The one-dimensional Davydov model is a crude approximation of the structure of a protein. Several authors have attempted to simulate solitons in proteins, including their three-dimensional features, using simplified (coarse-grained) classical^23^,^24^,^25^ or quantum dynamic models^26^. These models are very difficult to relate to actual protein dynamics. Protein dynamics span several time-scales in which local displacements of atoms are coupled to more large-scale conformational motions; a full description of these dynamics requires, therefore, an atomistic approach^16^.

In recent years, all-atom molecular dynamics (MD) simulations have become a powerful tool that is complementary to experiments to investigate the dynamics of proteins in solution at the atomic scale^27^,^28^,^29^. Here, we used all-atom MD simulations, including realistic interactions between water and amino acids and the full effects of temperature, to theoretically establish the lifetime and statistics of ILMs in proteins.

We predict a new type of fully classical ILMs in proteins: solitons localized in both time and space (similarly to the Peregrine solitons^30^,^31^). These intermittent ILMs are due to the anharmonicity of the potential energy surface describing the torsional degrees of freedom of the main chain of proteins. The torsional degrees of freedom play an important role in protein dynamics because they govern the low-frequency functional modes of proteins^32^,^33^,^34^. The main-chain torsional angles *γ* are built from four C^*α*^ atoms of consecutive residues in the amino acid sequence^35^,^36^ (Fig. 1). Because the length of the C^*α*^-C^*α*^ virtual bond between two consecutive residues is nearly constant, the main-chain conformation is entirely described by the main-chain torsional angles *γ* and the main-chain bond angles *θ* (Fig. 1). These coarse-grained angles (CGA) (*γ*, *θ*) are part of coarse-grained protein models^37^,^38^ and are used to analyze large conformational changes of proteins and protein folding in all-atom MD simulations^36^,^39^.

The origin of ILMs found in proteins can be better understood by drawing an analogy between the fluctuations of the protein main chain and those of a simple mechanical system known to substend solitons. The mechanical analog of the protein main chain is a chain of rigid pendulums coupled by harmonic torsional springs and rotating around the same axis^2^ [Fig. 2(a)]. At each time, the position of each pendulum *i* is defined by its angle *α*_*i*_ relative to the vertical. In this mechanical model, anharmonicity arises from the gravitational restoring force, which is proportional to sin(*α*_*i*_). This mechanical system may subtend ILMs (sine-Gordon solitons)^2^, which interpolate between two rest states (no restoring force) of the system (*α*_*i*_ = 0 for all *i* and *α*_*i*_ = 2*π* for all *i*). The rest state *α*_*i*_ = 0 for all *i* and the ILM solution of the dynamic equations^2^, computed at the maximum of its amplitude (*α* = *π*) for a localization chosen at *i* = 10, are shown in Fig. 2(a). The localized wave is characterized by a well-defined profile of the angle *α* [Fig. 2(b)] named *kink*. Due to the symmetry of the system, an anti-*kink* solution exists. The combination of the *kink* and anti-*kink* solutions may lead to a localized solution [chosen at *i* = 10 in Fig. 2(b)], that does not propage but does oscillate as a function of time, a so-called sine-Gordon discrete breather^2^. The position of each pendulum can be represented alternatively as a rotating unit vector in a plane: **u**_**i**_ = (cos(*α*_*i*_), sin(*α*_*i*_)) [Fig. 2(c)]. For each pendulum *i*, we define the difference between the vector **u**_**i**_ between the uniform state of the chain [red arrows in Fig. 2(c)] and in its excited state [blue arrows in Fig. 2(c)] by Δ**u**_**i**_. The kink [panel (b)] leads to a peak in 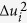 as a function of the pendulum position *i* [panel (d)] (in the example shown in Fig. 2, the sine-Gordon soliton is centered at *i* = 10). Consequently, the time-dependent fluctuations of the pendulums can be viewed as an ensemble of vectors rotating in a plane [Fig. 2(c)]. Their displacements between different times allow the definition of the localized character of the excitation [Fig. 2(d)].

As for the motion of each pendulum, the dynamics of each torsional angle *γ*_*i*_ of the protein main chain can be represented by a unit vector **u**_**i**_ = (cos(*γ*_*i*_), sin(*γ*_*i*_)) rotating in a plane^36^,^40^. For small fluctuations around their equilibrium orientation, the vectors are harmonically coupled, as are the pendulums in the mechanical model. However, for large angular displacements, anharmonicity arises due to the nonlinearity of the dihedral potential energy surface. As for the pendulums, the orientation of each vector **u**_**i**_ corresponding at *γ*_*i*_ = 0 and *γ*_*i*_ = 2*π* are equivalent, and solitons similar to discrete breathers of sine-Gordon type are expected in proteins by analogy.

More generally, the fluctuations of each pair of CGAs in the protein can be represented by a unit vector:


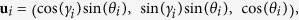


with one end fixed and the other describing a stochastic path along the surface of a sphere [Fig. 1(b)]. An example of the vectors **u**_**i**_ built on (*γ*_*i*_, *θ*_*i*_) (Fig. 1) computed from an all-atom MD trajectory of Trp-cage protein^41^ (see the Results section) are shown for a uniform state (red arrows) and an excited state (blue arrows) occuring 1 ps later at *i* = 10 [Fig. 2(c)]. The localized character of this particular excitation can be seen in Fig. 2(d), where the 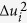 values are compared for the pendulum model and the protein. As seen in Fig. 2(c), the vector displacements are primarily due to the fluctuations of the *γ* torsional angles. As shown in the Results section, the typical ILM shown in Fig. 2(d) is a rare event in MD simulations of proteins and is localized in both time and space.

In the present work, we present evidence of ILMs of the soliton type [as in Fig. 2(d)] in the spontaneous, unbiased fluctuations of the main chain of model proteins at different temperatures using all-atom MD in explicit solvent (water). We predict the existence, statistics, and biophysical properties of ILMs in proteins and their relation with the protein free-energy landscape. The particular questions we address are as follows: what is the probability of finding an ILM occurring spontaneously in the native (folded) and non-native (misfolded or unfolded) states of a protein in solution? How do the ILMs depend on the secondary structures of the protein and on its chemical composition?

## Results

### Evidence of ILMs in unbiased MD simulations

As the loss of rigidity due to the unfolding of a protein increases the anharmonicity of its free-energy landscape, we investigated the dynamics of two ultrafast-folder proteins, Trp-cage^41^,^42^ and the chicken villin headpiece fragment HP-36^43^,^44^, above their folding temperature. These proteins were chosen because they have been extensively studied using MD simulations and experiments and because unfolding events can be reproduced by unbiased all-atom MD simulations in explicit solvent within a reasonable computational time^45^,^46^,^47^,^48^. Trp-cage is a 20-residue protein designed to aid in understanding protein folding mechanisms consisting of one *α*-helix and one (3/10)-helix^41^. HP-36 is a 36-residue protein corresponding to the C terminus of the 76-residue chicken villin headpiece domain^43^,^44^. It consists of three *α*-helices. Because of their small size and fast kinetics, Trp-cage and HP-36 have become typical model proteins for MD simulations of protein folding^45^,^46^,^47^,^48^. For comparison, we analyzed also the dynamics of a rigid 46-residue *α*/*β* model protein (VA3)^40^. Because of the presence of three disulfide bonds (namely 3–40, 4–32, and 16–26), VA3 remains folded in all MD trajectories^40^ while exploring the non-native state at 380 K.

**Figure 1:**
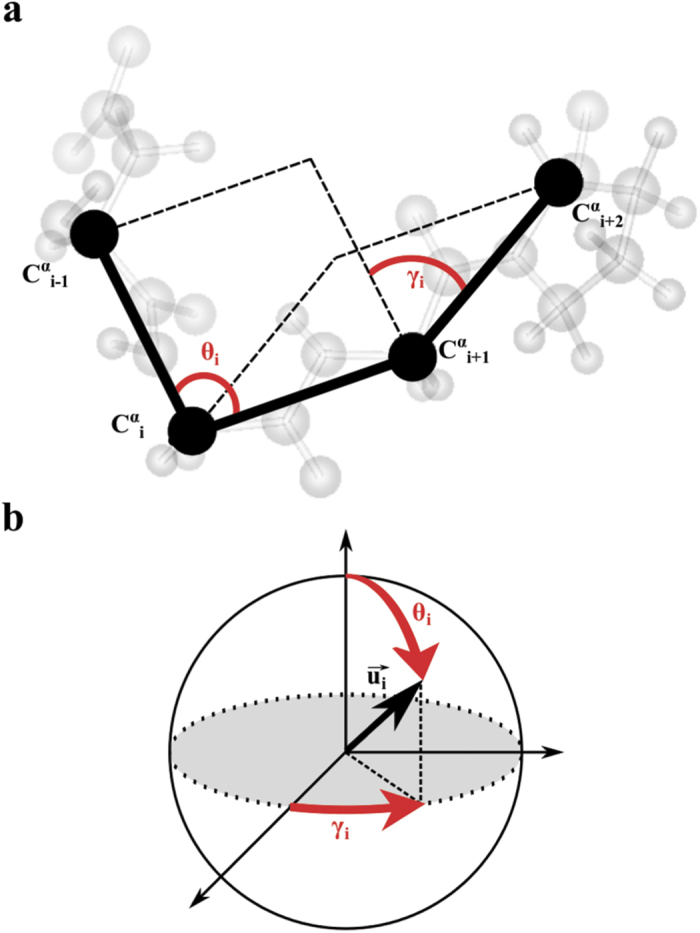
Coarse-grained angles (CGA) and their vector representation. (**a**) For a residue *i*, *γ*_*i*_ is the dihedral angle formed by the virtual bonds joining four successive C^*α*^ atoms (*i* − 1, *i*, *i* + 1 and *i* + 2) along the amino acid sequence and *θ*_*i*_ is the bond angle formed by the virtual bonds joining three successive C^*α*^ atoms (*i* − 1, *i* and *i* + 1) along the amino acid sequence. The first pair of CGA (*γ*, *θ*) along the sequence is (*γ*, *θ*)_2_ and the last one is CGA (*γ*, *θ*)_*N*−2_, where *N* is the total number of residues. The convention for *γ* angles is the following: each *γ* angle varies between −180° and +180° with *γ* = 0° being chosen when 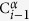 is cis to 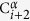, and the clockwise rotation of 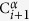-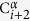 is positive when looking from 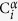 to 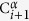. (**b**) Representation of the CGA pair (*γ*_*i*_, *θ*_*i*_) by a unit vector **u**_**i**_ in spherical coordinates, where *γ*_*i*_ is the azimuth angle, and *θ*_*i*_ is the polar angle.

**Figure 2:**
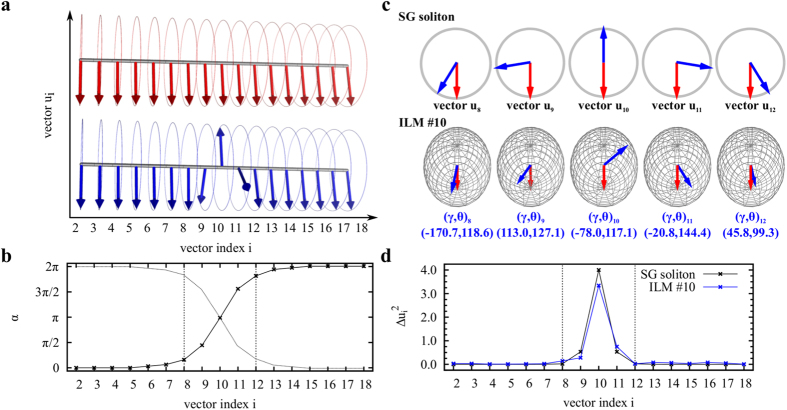
Analogy between solitons of a mechanical model and ILMs computed in proteins. (**a**) System of 17 coupled pendulums in its initial configuration (red) and in its excited state (blue). (**b**) Values of the angle *α*_*i*_ of each pendulum *i* relative to the vertical in the *kink* excited state [blue pendulums in panel (**a**)]. For comparison an anti-*kink* is shown using dashed lines. The dotted lines indicate the region of maximum localization. (**c**) (top) Vectors **u**_*i*_ = (cos(*α*_*i*_), sin(*α*_*i*_)) computed for the initial (red) and excited (blue) states of panel (**a**) for the region of maximum localization. (bottom) Vectors **u**_*i*_ = (cos(*γ*_*i*_)sin(*θ*_*i*_), sin(*γ*_*i*_)sin(*θ*_*i*_), cos(*θ*_*i*_)) extracted from an MD simulation of Trp-cage for an initial state (red) and excited state (blue). For more clarity, each initial vector (in red) has been positioned in the same configuration for all spheres by rotation, and the exact values of the initial state (*γ*_*i*_, *θ*_*i*_) are given. (**d**) Amplitude 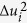 of a sine-Gordon soliton (black line) and the ILM extracted from MD simulations (blue line).

Three all-atom MD trajectories with different initial conditions (run 1, run 2 and run 3) at *T* = 380 K and one MD trajectory at *T* = 300 K each of a duration of 500 ns were conducted for Trp-cage, and two all-atom MD trajectories with different initial conditions (run 1 and run 2) at *T* = 380 K and one MD trajectory a *T* = 300 K each of a duration of 500 ns were conducted for HP-36 in explicit water (see the Methods section). In addition, one all-atom MD trajectory at 300 K and one at 380 K each of a duration of 500 ns were performed for VA3 (see the Methods section). The coordinates of the proteins were recorded every ps. Each MD run, therefore, represents 500,001 snapshots, from which the vector **u**_**i**_ associated with each pair (*γ*_*i*_, *θ*_*i*_) (Fig. 1) was computed. The fluctuations of the protein main chain between two consecutive snapshots were represented by the sequence of the displacements Δ**u**_*i*_(*t*) = **u**_*i*_(*t*) − **u**_*i*_(*t* − 1) for all *i* = 2 to *N* − 2 and *t* ≤ 1. The degree of localization of these fluctuations was measured by the normalized Shannon entropy *S* computed from the square displacements Δ*u*_*i*_(*t*)^2^ along the sequence (see the Methods section). An excitation localized on a single pair of CGAs corresponds to *S* = 0 (minimum entropy, strongly localized fluctuations), and an excitation uniformly distributed on all CGAs corresponds to *S* = 1 (maximum entropy, delocalized fluctuations). The calculation of *S*(*t*) is a systematic means to detect rare large localized excitations in MD trajectories. The ILMs are defined here by the excitations for which *S* ≤ 0.5 (see the Methods section for the choice of this cutoff value). For example, the ILM shown in Fig. 2(d), which is the excitation that has the largest amplitude 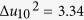 in the MD run 1 (*T* = 380 K) of Trp-cage, had a value of *S* = 0.47. The results for the Trp-cage protein are discussed next, and similar results for additional MD runs of Trp-cage, HP-36 and VA3 are shown in the Supplementary Information.

**Figure 3:**
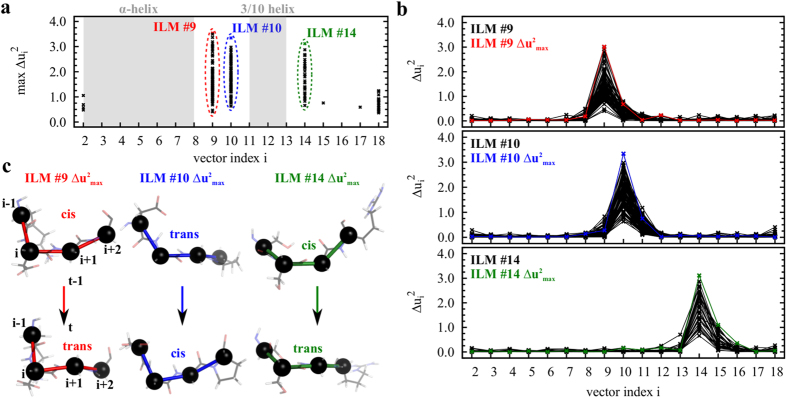
Intrinsic localized modes (ILMs) for the Trp-cage protein at *T* = 380 K. Results of the MD run 1 are shown. (**a**) Maximum amplitudes Δ*u*^2^ of ILMs as a function of the residue index *i*. (**b**) Amplitudes Δ*u*^2^ of ILMs *i* = 9, 10 and 14 as a function of the vector index. The ILMs showing the largest amplitude are highlighted in red (ILM #9), blue (ILM #10) and green lines (ILM #14). (**c**) Typical cis-trans and trans-cis transitions observed in ILMs #9, #10 and #14 of maximum amplitude.

ILMs (*S* ≤ 0.5) are rare events. For example, in the MD run 1 of Trp-cage, only 251 ILMs were found (Table 1), which represents 0.05% of the total number of main-chain fluctuations recorded over 500 ns. The probability of observing ILMs was similar in the other MD runs of Trp-cage (Table 1). In all MD runs of Trp-cage, the most frequent ILMs were located at the same specific positions along the amino acid sequence [Fig. 3(a)]: *i* = 9, 10, 14 and 18. The excitations at *i* = 9, 10 and 14 are typical solitons of sine-Gordon type [compare Fig. 2(d) to Fig. 3(b)]. The largest amplitudes of ILMs were found for these three sites, with Δ*u*^2^ = 3.0, 3.3 and 3.1 for a soliton centered at *i* = 9, 10 and 14, respectively. Most of the solitons (80%) centered at *i* = 9, 10 and 14 with the largest amplitudes (Δ*u*^2^ > 2.0) corresponded to cis-trans or trans-cis transitions of the four C^*α*^ segments [typical examples are shown in Fig. 3(c)].

The solitons localized at *i* = 9, 10 and 14 [shown in Fig. 3] shared a common feature: they all had a glycine (GLY) residue at *i* + 1 in the amino acid sequence (in bold font in Table 1). As shown in SI (Supplementary Tables 1 and 2), similar results were found for HP-36 and VA3 proteins: solitons localized at *i* = 51 and *i* = 73 in HP-36 have a GLY residue at *i* = 52 and at *i* = 74, respectively, and solitons localized at *i* = 36 in VA3, have a GLY residue at *i* = 37. In the Trp-cage, the most frequent soliton (*i* = 10) corresponded to a rotation around a virtual bond formed by two GLYs (GLY10-GLY11) (Table 1). Because of its small side-chain (H atom), GLY can adopt a larger set of conformations in a polypeptide chain, which may explain why the highest probabilities of ILMs are observed at *i* = 9, 10 and 14. To test this hypothesis, we ran an MD trajectory for the mutant Trp-cage G15A at *T* = 380 K. The number of ILMs of soliton type at *i* = 14 decreases by an order of magnitude (Table 1). To further test the role of GLYs, we ran a MD trajectory for the triple mutant Trp-cage G10A-G11A-G15A at *T* = 380 K. The number of ILMs of soliton type at *i* = 9, 10 and 14 decreased drastically (Table 1). This observation reveals, for the first time, the role of GLY residues in the localization and the probability of the appearance of ILMs of the soliton type.

The ILMs located at the C-termimus of the chain (*i* = 18) are *not* similar to sine-Gordon solitons (Supplementary Figure 1) but are localized excitations that also exist in a harmonic chain with free ends and are due to the broken symmetry of the chain at its extremities^49^. These ILMs do not depend on the presence of GLY residues, and their probability is similar for Trp-cage and its mutants (Table 1).

A few ILMs (*S* ≤ 0.5), all with small amplitudes (0.4 < Δ*u*^2^ < 1.9), were also observed very rarely (not more than six times in 500,001 snapshots) at *i* = 2, 7, 8, 11, 12, 13, 15, 16 and 17 in the MD runs of Trp-cage (Supplementary Table 3). Except at *i* = 2, which corresponds to a mode located at the N-terminus (Supplementary Figure 1), all of these ILMs were similar to sine-Gordon solitons.

### Free-energy landscape and ILMs

The probability of observing an ILM at a given time is smaller if the protein is in its native state (rigid, folded state) than in a non-native state (flexible, misfolded or unfolded states). The native state of Trp-cage is defined by the ensemble of the most probable conformations explored at *T* = 300 K. The native state is better represented as basins in the free-energy landscape of the protein^6^,^16^.

We represented the free-energy landscape of the main chain of Trp-cage by the sequence of the effective free-energy maps *V*(*γ*, *θ*)_*n*_ computed from the probability densities of each pair of CGA (*γ*, *θ*)_*n*_ in the MD trajectories (see the Methods section and Supplementary Figures 2 and 3). The sequence of *V*(*γ*, *θ*)_*n*_ has proven useful in describing protein folding^50^, conformation dynamics^40^ and allosteric communication^51^. For each *V*(*γ*, *θ*)_*n*_, we defined the native basin as the region of the (*γ*, *θ*)_*n*_ space within 3 k_*B*_T from the minimum of *V*(*γ*, *θ*)_*n*_ at *T* = 300 K (Supplementary Figure 2). The 3 k_*B*_T cutoff ensures that all of the experimental structures of Trp-cage observed by NMR at 282 K^41^ correspond to (*γ*, *θ*) angles located in the native basins (Supplementary Figure 4).

**Figure 4:**
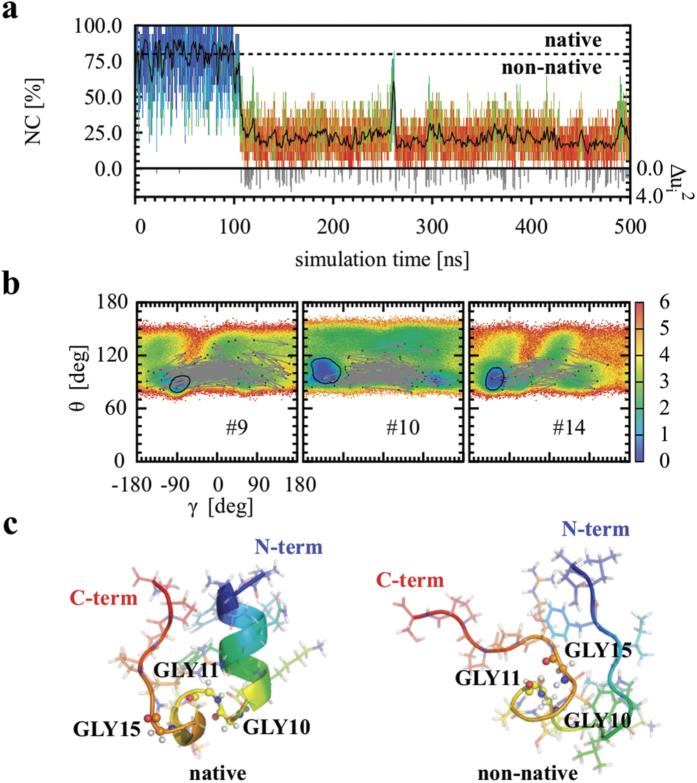
Relation between ILMs and the native/non-native character of the Trp cage protein (run 1 is shown). (**a**) Native character (*NC*) (in %) as a function of simulation time. The color line using the BGR palette (from blue, which corresponds to *NC* = 100%, green, which corresponds to *NC* = 50%, to red, which corresponds to *NC* = 0%) represents the NC computed every 1 ps, and the black line represents the mobile average of the NC computed in a time window of 1 ns. Gray impulses shown on the time axis of the lower graph represent the occurrence of an ILM (*S* ≤ 0.5) along the MD trajectory. (**b**) Effective free-energy map *V*(*γ*, *θ*)_*n*_ (kbT units) for (*γ*, *θ*)_9_ (left panel), (*γ*, *θ*)_10_ (middle panel) and (*γ*, *θ*)_14_ (right panel). Black lines represent free-energy isolines *V* − *V*_*min*_ = 3 k_*B*_T computed from the effective free-energy maps *V*(*γ*, *θ*)_*n*_ at *T* = 300 K (Supplementary Figure 2). Vectors in gray lines represent the displacements on the map for all the corresponding ILMs. (**c**) Structures of the Trp cage in typical native (left panel) and non-native (right panel) conformations. Structures are shown in a cartoon plus stick representation. GLY residues are highlighted in ball and stick representations. The color code corresponds to an RGB palette, from N-terminus (residue 1) to C-terminus (residue 20).

To quantify the native character (*NC*) of the protein as a function of time in MD trajectories at *T* = 380 K, we counted the % of CGA remaining within their native basins, as illustrated in Fig. 4(a), for a typical trajectory. At *T* = 380 K, Trp-cage partially folds/unfolds during MD runs, *i.e.*, explores non-native states far from the native basins of the free-energy maps, as shown for selected CGA pairs in Fig. 4(b) and for all of the CGAs in Supplementary Figure 3. Typical structures of the protein in native and non-native states are shown in Fig. 4(c) and in the Supplementary Figure 5 for another MD trajectory (run 2) for comparison. As shown in Fig. 4(a), a sharp transition occurs between the initial native state of Trp-cage, which lasts for approximately 100 ns, to a non-native state in which the molecule remains until the end of the trajectory [Fig. 4(a)]. Results similar to those presented in Fig. 4(a) were found for all the MD trajectories of Trp-cage and HP-36 at *T* = 380 K (with different sequences of folding and unfolding events depending on the initial conditions of the MD runs, see Supplementary Figures 5 and 6). As clearly shown in Fig. 4 [bottom of panel (a)], the number of ILMs was larger in the non-native portion of the trajectory than in its native portion. The same results were observed for all MD runs of Trp-cage, HP-36 and VA3 (Supplementary Figures 5 and 6) and were quantified by computing the probability of observing a soliton in the native portion and in the non-native portion of the trajectory (Table 2). For the fast-folder proteins examined in the present work, Table 2 demonstrates that the probability of observing a soliton is larger by a factor varying between approximately 9 and 28 in the non-native portion of the trajectory compared to the native portion at *T* = 380 K. The probability of observing a soliton is only about twice larger in the non-native portion of the MD trajectory of VA3 than in the native portion of the trajectory at *T* = 380 K (Table 2). Because of its three disulfide bridges, VA3 did not unfold in the MD run and explored a non-native state with a relative high average of *NC* (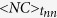 = 75%, see Supplementary Figure 6). In reference to Table 2, it is worth noting that the probability of observing a soliton in the non-native portion of the trajectories at *T* = 300 K is difficult to evaluate accurately, as the time spent in a non-native state is extremely small.

### Statistics of ILMs at different time resolutions

**Figure 5:**
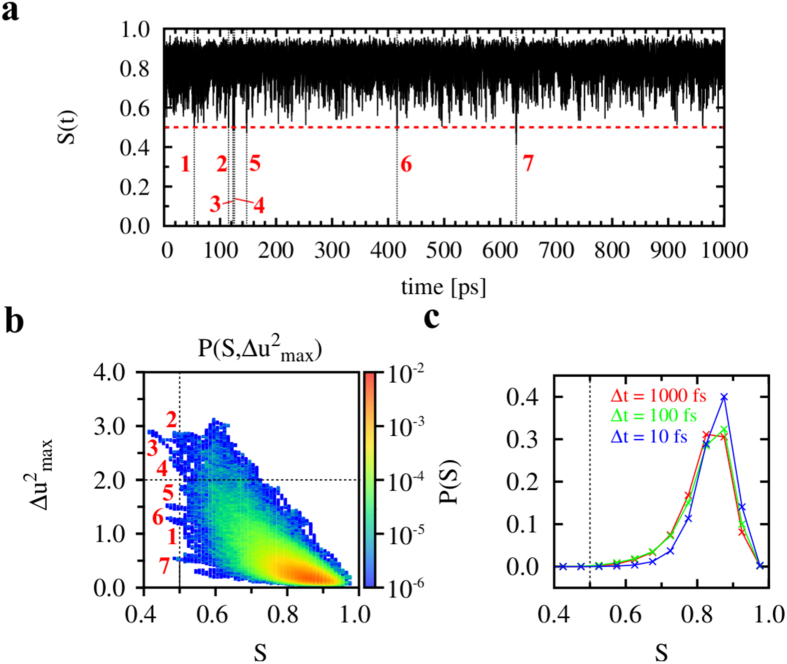
Statistical analysis of ILMs at different time resolutions. (**a**) Shannon entropy *S* as a function of simulation time computed every 1 fs from a typical 1 ns MD run of Trp-cage at *T* = 380 K. The black dashed lines indicate ILMs (*S* ≤ 0.5), and the red dashed line indicates the criterion *S* = 0.5. (**b**) 2-D probability distribution function 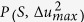 computed from the same MD run of Trp-cage at 380 K as in panel a. The horizontal and vertical black dashed lines indicate the limits 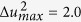 and *S* = 0.5, respectively. (**c**) Probability distribution function *P*(*S*) computed using different time resolution using the same trajectory as in panels a and b.

To improve the statistics of ILMs, we ran eight additional short trajectories of 1 ns duration using initial non-native structures of Trp-cage extracted at different times from MD run 1 at *T* = 380 K and recording a snapshot every fs. The displacements of the main chain, Δ**u**_*i*_, were first computed from these trajectories by using a sliding window of Δ*t* = 1 ps shifted every fs. That is, the time-scale of the displacements Δ*u*_*i*_ was identical to that discussed in the previous sections. The ILMs of the soliton type similar to those reported in Fig. 3(b) were detected by computing *S*(*t*) every fs [see Fig. 5(a)]. Seven solitons were detected [Fig. 5(a)] at *i* = 10 (solitons 1 to 5); *i* = 3 (soliton 6) and *i* = 14 (soliton 7) (not shown). Soliton 3 had the largest amplitude and was the most localized [Fig. 5(b)]. The probability of observing solitons in these short trajectories was similar to that reported in Table 1. If the snapshots were recorded only every ps (as in the 500 ns MD runs 1, 2 and 3), only soliton 3 (located at *i* = 10) would have been detected. Interestingly, the probability density of the entropy *P*(*S*) was fairly independent of the time-scale used to compute the main-chain displacements Δ*u*_*i*_ [see Fig. 5(c) for Δ*t* = 100 fs and Δ*t* = 10 fs]. The ILMs (*S* ≤ 0.5) were always found in the tails of *P*(*S*) (rare events).

### Soliton dynamics at a femtosecond time resolution

**Figure 6:**
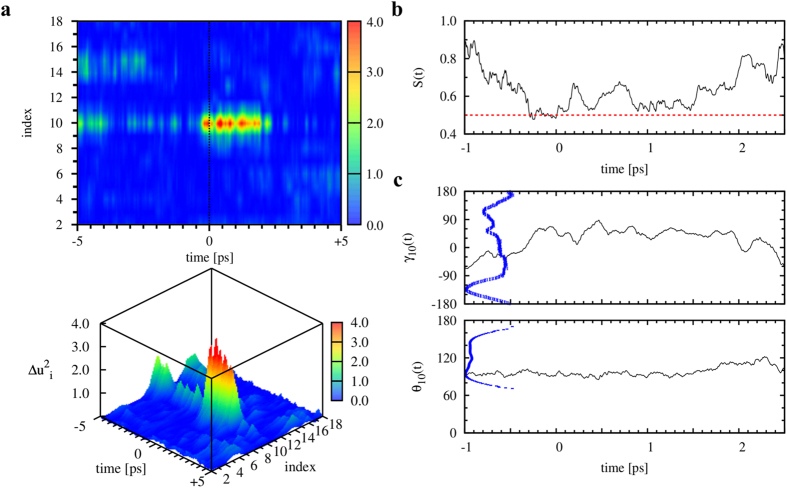
Typical time evolution of an ILM of soliton type in MD trajectories of Trp-cage of 1 ns duration for which the coordinates were recorded every fs. The time *t* = 0 corresponds to the detection of soliton 3 in the MD trajectory shown in Fig. 5. (**a**) Two-dimensional (up) and three-dimensional (bottom) color maps of 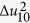 in a window of 10 ps. (**b**) Corresponding variation of *S*(*t*) between *t* = −1 ps and *t* = 2.5 ps. (**c**) Variations of (*γ*, *θ*)_10_ (thin black lines) in the time window represented in (**b**). The free-energy profiles *V*(*γ*_10_) and *V*(*θ*_10_) are also shown in thick blue lines. A movie of the time evolution of the soliton shown is available in the SI.

As an example, we analyzed the dynamics of soliton 3 (located at *i* = 10) detected in the trajectory shown in Fig. 5(a), for which snapshots were recorded every fs. The time *t* = 0 was defined as the time at which the soliton was detected, with Δ*t* = 1 ps in the trajectory analyzed in Fig. 5(a). The displacement Δ**u**_10_(*t*) was computed at each fs by summing the displacements Δ**u**_10_ every fs from *t* = −5 ps to *t* = 5 ps (by assuming Δ**u**_10_(*t* = −5 ps) ≡ 0). The values of Δ*u*_10_(*t*)^2^ shown in Fig. 6(a), demonstrated that the ILM is strongly localized between *t* = −1 ps and *t* = 2.5 ps. A careful analysis of the variation of the *S*(*t*) [Fig. 6(b)] shows that the life time of the soliton (*i.e.*, the time for which *S*(*t*) remains lower or close to 0.5) is only 400 fs (from *t* = −300 fs to *t* = 100 fs). This extreme time-space localization corresponds to a jump in the *γ*_10_ free-energy profile from the top of a barrier (*γ*_10_ = −30°) to a small metastable state (*γ*_10_ = 60°) [Fig. 6(c)]. As already noted in the introduction and shown in Fig. 2, the ILMs of the soliton-type correspond mainly to a large variation of the dihedral angle *γ*, as seen for soliton 3 in Fig. 6(c).

## Discussion

In the present all-atom MD study, ILMs were successfully detected in the spontaneous thermal fluctuations of the main-chain of proteins. We found that ILMs of soliton type are short-life events. In the example detailed in Fig. 6, the lifetime is ~400 fs (*S* ≤ 0.5) [it would be 1,500 fs if we adopted a less strict definition of the spatial localization of the main-chain deformation (*S* ≤ 0.6)]. These sub-picosecond localized excitations may be accessible for experimental investigation using 2D-IR spectroscopy, as demonstrated for the fast dynamics of the Ramachandran dihedral angular fluctuations of a three-peptide chain^52^. The probability of an ILM is approximately 0.05% in MD simulations. As rare events, these ILMs do not contribute significantly to the partition function of the system; they are a priori negligible from a thermodynamic equilibrium point of view. However, the sine-Gordon localized excitations may help to cross an activation barrier as illustrated in Fig. 6(c) and may be important from a kinetic point of view. Intermittent events often do play a role in biological function, for example, in conformational gating of a ligand in enzymes^53^.

Solitons were previously predicted to occur in stiff regions of a protein (in an *α*-helix or at catalytic sites) using simplified models of a polypeptide chain^23^. The opposite is found here by using all-atom MD simulations of two *α*-helical fast-folder proteins and a rigid protein. We found ILMs in the *flexible* regions of the proteins (loops and N- and C-termini), as illustrated in Fig. 3(a). These ILMs are different from the usual classical discrete breathers as they are strongly localized in time. Solitons with the highest amplitudes mainly corresponded to (incomplete) cis-trans or trans-cis transitions [Fig. 3(c)], which are more probable in flexible segments of a protein main chain. The flexibility governs the probability of observing a soliton: the probability of observing an ILM of the soliton type [Fig. 2(d)] is enhanced in the non-native state compared to that in the native state and in protein segments containing GLY residues. This result is expected because the main chain experiences more of the anharmonic portion of the free-energy landscape in large fluctuations than it does in small fluctuations. The non-native state was significantly explored at *T* = 380 K. To confirm that the solitons occur more frequently in our simulations because the protein is unstructured (and is thus more flexible) and not because the temperature is increased, we ran an additional 20 ns trajectory at *T* = 300 K by selecting one of the most unfolded structures of Trp-cage as the initial structure of the new trajectory, as we did previously^39^. The probability of observing solitons was enhanced by a factor of approximately 6 compared to that of the native state at the same temperature (Table 1). Therefore, ILMs of the soliton type might play a functional role in misfolded proteins and in unfolded proteins.

## Methods

### MD simulations

All-atom MD simulations in explicit water (TIP3P force field^54^) with Trp-cage (model 1 in PDB ID: 1L2Y)^41^, the chicken villin headpiece subdomain HP-36 (model 1 in PDB ID: 1VII)^44^ and the protein VA3 (model 1 in PDB: 1ED0)^55^ were conducted using the GROMACS software package^56^ and the AMBER99sb-ILDN force field^57^. In addition, one all-atom MD run of the Trp-cage at *T* = 300 K and one at 380 K using CHARMM27^58^,^59^ and AMBER99SB*-ILDN-q^60^ force fields were conducted. The results found using these force fields are reported in the Supplementary Table 4 and were similar to those obtained with AMBER99sb-ILDN force field. The time step used in all simulations was 1 fs, and the list of neighbors was updated every 5 fs with the grid method and a cutoff radius of 1.0 nm. The coordinates of all the atoms in the simulation box were saved every 1 ps. The initial velocities were chosen randomly. We used the NPT ensemble with a cubic box of 4.55 nm for Trp-cage, 5.21 nm for HP-36 and 5.27 nm for VA3. The temperature and pressure were kept to the desired value by using the Nosé-Hoover thermostat^61^,^62^ and the Parrinello-Rahman^63^ barostat, respectively. The electrostatic term was computed by using the particle mesh Ewald algorithm^64^ (with a radius of 1 nm) using the Fast Fourier Transform optimization (with an order equal to four for the interpolation). The cutoff algorithm was applied for the non-coulomb potentials with a radius of 1.0 nm. The system was warmed up for 50 ps and equilibrated for 1 ns with lower restraints, finishing with no restraints at the desired temperature. We performed three MD runs of Trp-cage and two MD runs of HP-36 at *T* = 380 K using different initial conditions and one MD run at *T* = 300 K for each protein (named run 1 in Table 1). In addition, we performed one MD run for each mutant of the Trp-cage ([G15A] and [G10A-G11A-G15A]) and for VA3 at *T* = 300 K and one at *T* = 380 K. Each MD run was of 500 ns duration. In total, the thirteen long MD runs corresponded to 6.5 *μ*s of simulations.

In addition, we performed eight extra short MD runs of Trp-cage of 1 ns duration at *T* = 380 K using the same procedure as described above, except that the coordinates of all the atoms in the simulation box were saved every 1 fs. The initial structures of those MD runs were extracted at different times from the MD run 1 of Trp-cage at *T* = 380 K, corresponding to *t* = 100, 161, 209, 283, 300, 441 and 459 ns. Data from the MD run using the frame at *t* = 441 ns are presented in the present paper (Figs 5 and 6). The results for the other seven runs were similar. Finally, we performed an MD run of Trp-cage of 20 ns duration at *T* = 300 K (named run 2 in Table 1) using a completely unfolded structure of the protein obtained in a previous work^39^ at *T* = 450 K using the same procedure described above.

### Free-energy map and free-energy profiles

An effective free-energy map *V*((*γ*, *θ*)_*i*_) and two free-energy profiles *V*(*γ*_*i*_) and *V*(*θ*_*i*_) were computed for each pair of CGAs (*γ*, *θ*)_*i*_ by using


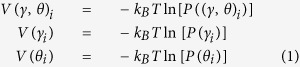


where *k*_*B*_ is the Boltzmann constant, *T* is the temperature and *P*((*γ*, *θ*)_*i*_), *P*(*γ*_*i*_), *P*(*θ*_*i*_) are the probability density functions (PDF) of the pair (*γ*, *θ*)_*i*_, of *γ*_*i*_ and of *θ*_*i*_, respectively. The PDFs were computed from the MD trajectories on a time-scale of 500 ns.

### Normalized Shannon entropy *S* and localized excitations

The quantity *p*_*i*_ measures the fluctuations of the pair of CGA *i* (*i* = 2 to *N* − 2) relative to the *N* − 3 pairs of CGAs along the sequence and is defined by


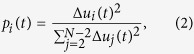


where Δ*u*_*i*_ is the displacement of the vector **u**_*i*_ between two consecutive snapshots Δ**u**_*i*_(*t*) = **u**_*i*_(*t*) − **u**_*i*_(*t* − 1) and **u**_*i*_ = (cos(*γ*_*i*_)sin(*θ*_*i*_), sin(*γ*_*i*_)sin(*θ*_*i*_), cos(*θ*_*i*_)). By definition, 0 ≤ *p*_*i*_(*t*) ≤ 1. The localization of the structural fluctuations of the protein main chain can be quantified by


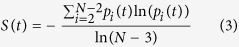


where *S*(*t*) can be interpreted as a normalized Shannon entropy 0 ≤ *S*(*t*) ≤ 1. The maximum localization of the fluctuations occured for *S* = 0 (*p*_*i*_ = 0 for *i* ≠ *k* and *p*_*k*_ = 1), and the maximum delocalization occured for *S* = 1 (*p*_*i*_ = 1/(*N* − 3) for all *i*).

An ILM is defined by a value of *S* ≤ *S*_*cutoff*_, where *S*_*cutoff*_ is chosen such that the correlation coefficient between the sequence of the Δ*u*_*i*_(*t*)^2^ of all ILMs localized at the same site *i* is larger than 0.9. For example, in the MD run 1 (*T* = 380 K), the most strongly localized excitation at *i* = 10 had a value of *S* = 0.40 with a maximum amplitude Δ*u*_10_(*t*)^2^ = 2.42. All excitations located at *i* = 10 are highly correlated (*ρ* > 0.9) if *S* ≤ 0.5, *i.e.*, they all represent the same single peak of localized excitation (Supplementary Figure 7). It is worth noting that the number of ILMs at *i* = 10 in this MD run increases as a function of the *S*_*cutoff*_ value (see Supplementary Table 5) as expected. However, the probability to find an ILM at *i* = 10 in the non-native state is larger than in the native state for different values of *S*_*cutoff*_. The ratio *P*_*nn*_/*P*_*n*_ is infinite for *S*_*cutoff*_ = 0.45 and decreases to 4.6 at *S*_*cutoff*_ = 0.6 (Supplementary Table 5) because at *S*_*cutoff*_ = 0.6 we include excitations which are not centered on a single site as the one shown in red in the Supplementary Figure 7. A similar conclusion was drawn for all of the MD runs studied here, which sets the value of *S*_*cutoff*_ to 0.5.

## Additional Information

**How to cite this article**: Nicolaï, A. *et al.* Intrinsic Localized Modes in Proteins. *Sci. Rep.* **5**, 18128; doi: 10.1038/srep18128 (2015).

## Supplementary Material

Supplementary Information

Supplementary Video

## Figures and Tables

**Table 1:** Number *N*_*ILM*_ of ILMs detected in MD simulations of Trp-cage at *T* = 380 K and 300 K.

	*T* = 380 K					*T* = 300 K	
	run 1	run 2	run 3	mutant^+^	mutant*	run 1	run 2
*N*_*ILM*_	251	302	199	321	58	71	17
*N*_*ILM*_/*N*_*t*_ (in %)	0.0502	0.0604	0.0398	0.0642	0.0116	0.0142	0.085
*i* = 9 (LYS-ASP-GLY*-GLY*)	95	92	42	167	2	—	—
*i* = 10 (ASP-GLY*-GLY*-PRO)	93	110	59	113	7	2	16
*i* = 14 (SER-SER-GLY^+,^*-ARG)	33	63	41	2	7	—	—
Solitons *i* = 9, 10 and 14	221	265	142	282	16	2	16
*i* = 18 (PRO-PRO-PRO-SER)	24	29	46	25	26	62	—

Localization along the amino acid sequence and statistics of the most frequent ILMs (*i* = 9, 10, 14 and 18) are given. For each mutant, each residue replaced by ALA is marked by a symbol. All MD runs were of a 500 ns duration except run 2, at *T* = 300 K (20 ns, see the Methods section).

**Table 2:** Statistics of ILMs of the soliton type in the native and non-native states computed from MD simulations of Trp-cage, chicken villin headpiece fragment HP-36, and VA3 (MD trajectories of a duration of 500 ns are presented).

Protein	*T* [K] (run)	*t*_*n*_ [ns]	*t*_*nn*_ [ns]	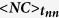	*N*_*n*_	*N*_*nn*_	*P*_*n*_	*P*_*nn*_	*P*_*nn*_/*P*_*n*_
Trp cage	300(1)	487	13	—	9	—	0.018	—	—
	380(1)	57	443	27%	1	222	0.018	0.501	27.8
	380(2)	11	489	26%	—	270	—	0.552	—
	380(3)	71	429	32%	1	149	0.014	0.347	24.6
HP-36	300(1)	478	22	—	28	3	0.059	0.136	2.3
	380(1)	64	436	58%	1	83	0.016	0.190	11.9
	380(2)	185	315	66%	4	58	0.022	0.184	8.4
VA3	300(1)	500	—	—	33	—	0.066	—	—
	380(1)	245	255	74%	12	22	0.049	0.086	1.8

The subscripts *n* and *nn* define the native and non-native states of the protein, respectively. ILMs at the N-terminus (localized at *i* = 2) and C-terminus (localized at *i* = 18, 34 and 44 for Trp-cage, HP-36 and VA3, respectively) were not considered.
