# Supplementary material for: Intrinsic Localized Modes in Proteins
Source: Sci Rep. 2015 Dec 11;5:18128. doi: 10.1038/srep18128 (PMC4676062; doi:10.1038/srep18128)
Supplement: Supplementary Information [file srep18128-s1.pdf]

Supplementary Information
Intrinsic Localized Modes in Proteins

Adrien Nicolaï[1,2], Patrice Delarue[2] and Patrick Senet[2,*]

[1] Department of Physics, Applied Physics and Astronomy, Rensselaer Polytechnic Institute, Troy, 12180 New York, United States.

[2] Laboratoire Interdisciplinaire Carnot de Bourgogne, UMR 6303 CNRS-Univ. Bourgogne Franche-Comté, 9 Av. A. Savary, BP 47 870, F-21078 Dijon Cedex, France.

[*] Corresponding author: psenet@u-bourgogne.fr.

List of Supplementary Figures:

- Figure 1
- Figure 2
- Figure 3
- Figure 4
- Figure 5
- Figure 6
- Figure 7

List of Supplementary Tables:

- Table 1
- Table 2
- Table 3
- Table 4
- Table 5

List of Supplementary Movies:

- Movie 1

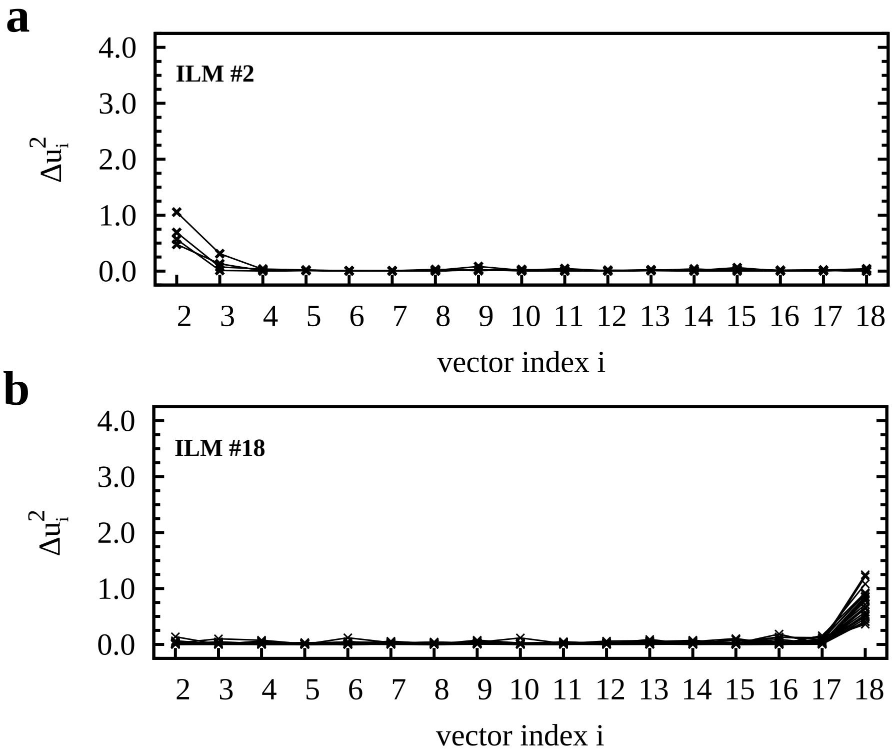

Supplementary Figure 1: Amplitudes $\Delta {u_i}^2$ of ILMs $i = 2$ and 18 as a function of vector index.

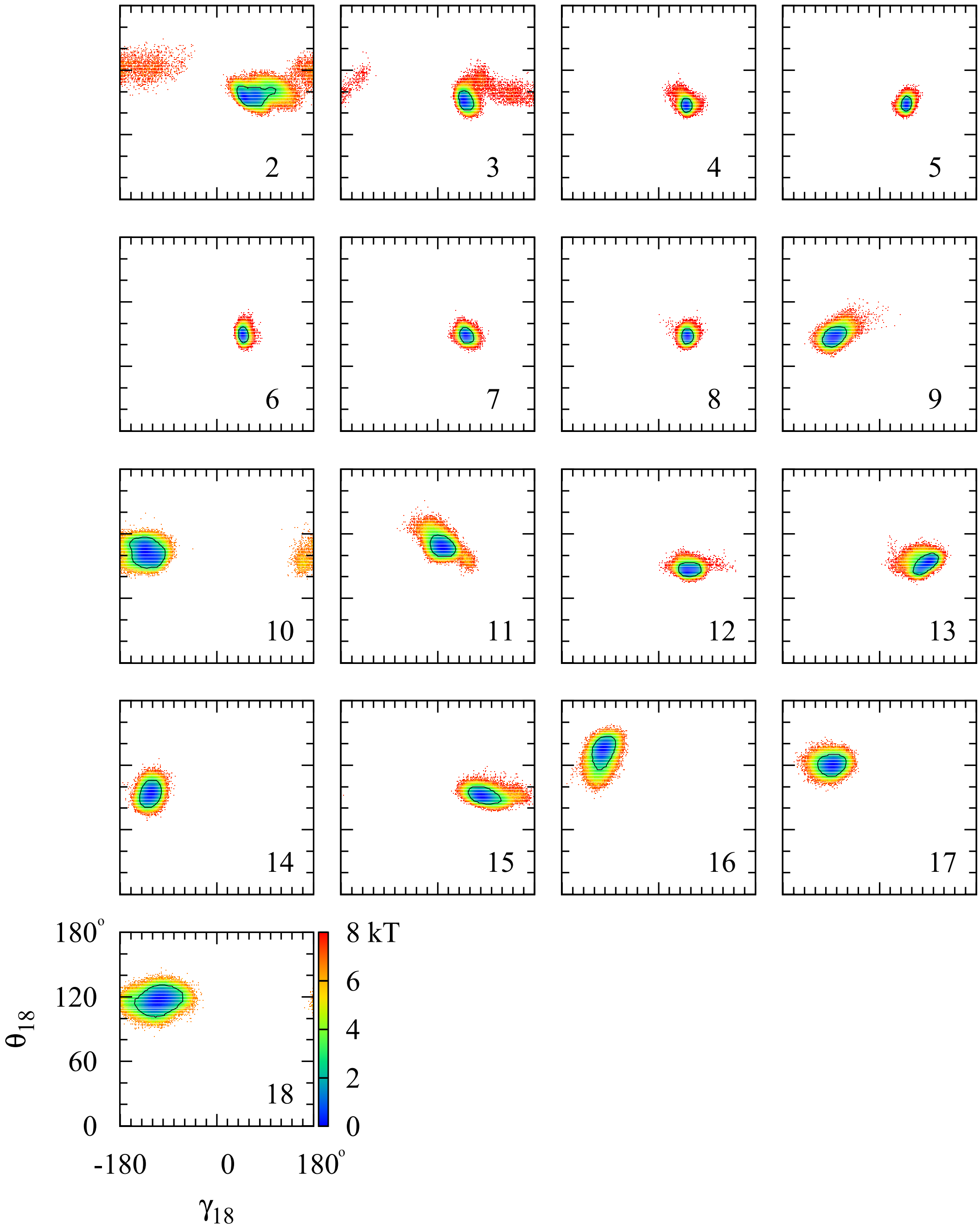

Supplementary Figure 2: Effective free-energy map $V(\gamma, \theta)_n$ for n =2 to 18 computed from MD run 1 of Trp-cage at T = 300 K. Black lines represent free-energy isolines $V - V_{min} = 3k_BT$.

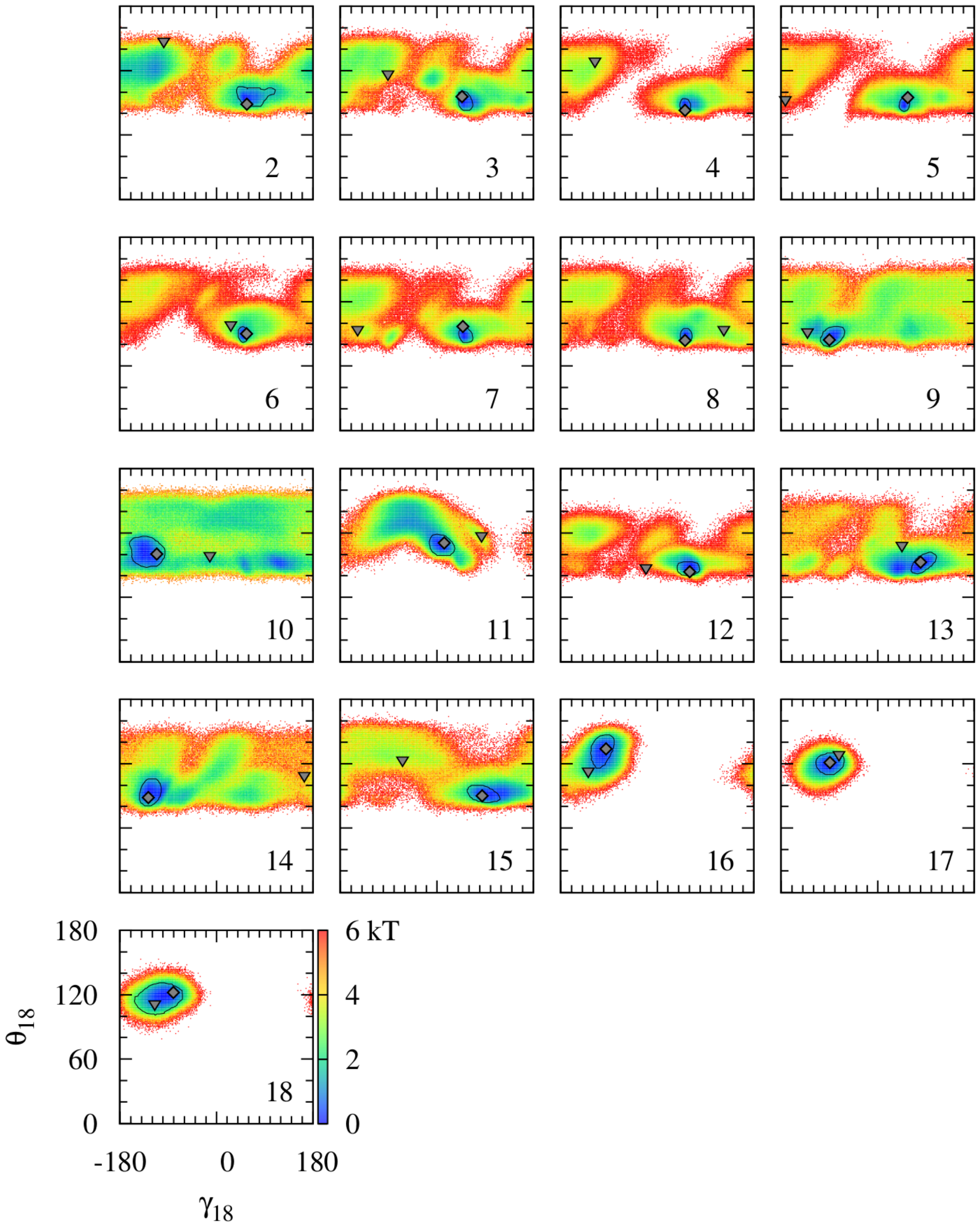

Supplementary Figure 3: Effective free-energy map $V(\gamma,\theta)_n$ for $n = 2$ to 18 computed from MD run 1 of Trp-cage at $T = 380$ K. Black lines represent free-energy isolines $V - V_{min} = 3k_B$T computed from the effective free-energy maps $V(\gamma,\theta)_n$ at $T = 300$ K (see Supplementary Figure 2). Gray diamond and triangle represent (γ,θ) values of typical native and non-native conformations of Trp-cage, respectively, as shown in Figure 4 (c).

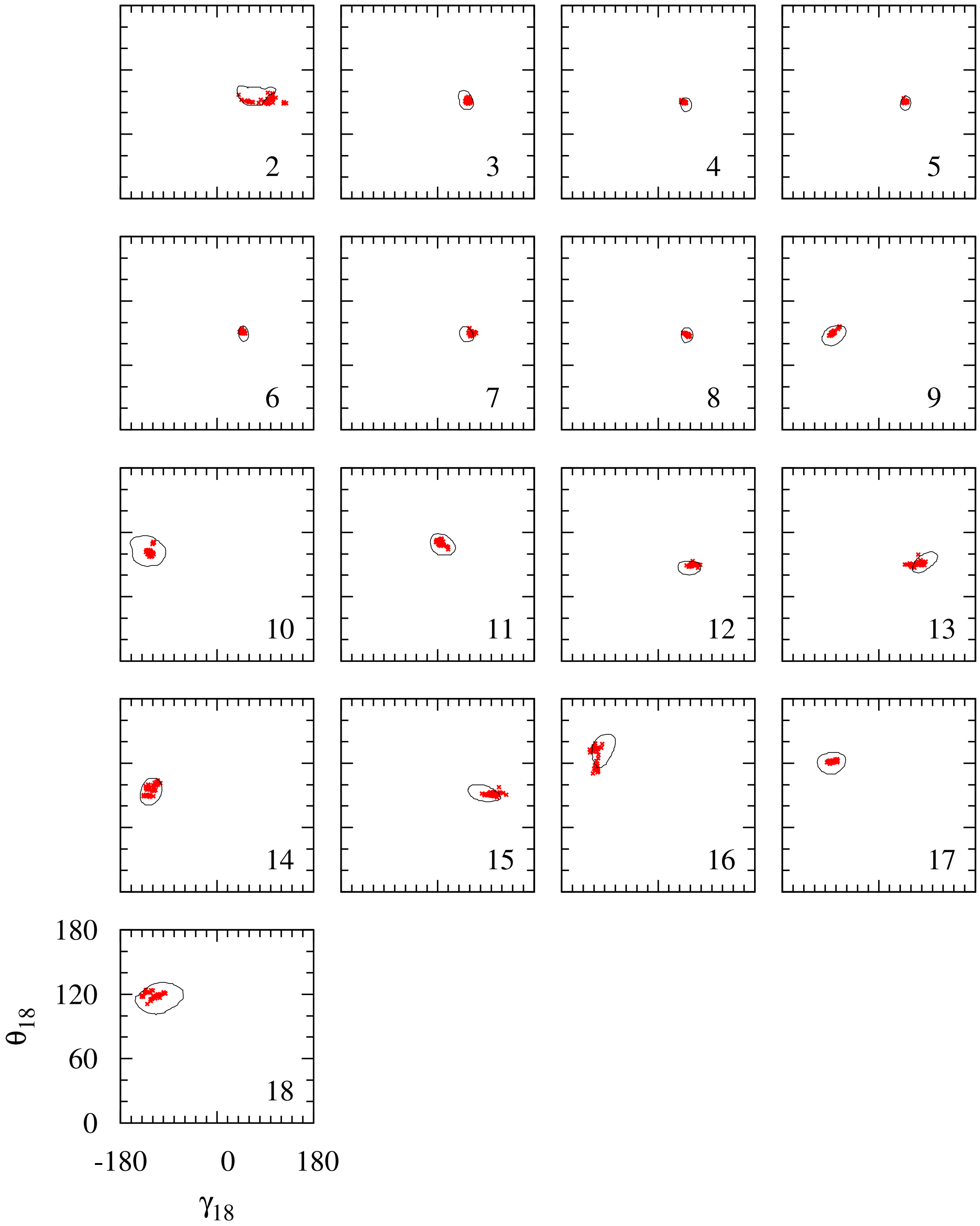

Supplementary Figure 4: $(\gamma, \theta)_n$ for $n = 2$ to 18 computed from NMR-derived structures provided in the PDB file 1L2Y. Black lines represent free-energy isolines $V - V_{min} = 3k_B T$ computed from the effective free-energy maps $V(\gamma, \theta)_n$ at $T = 300$ K (see Supplementary Figure 2).

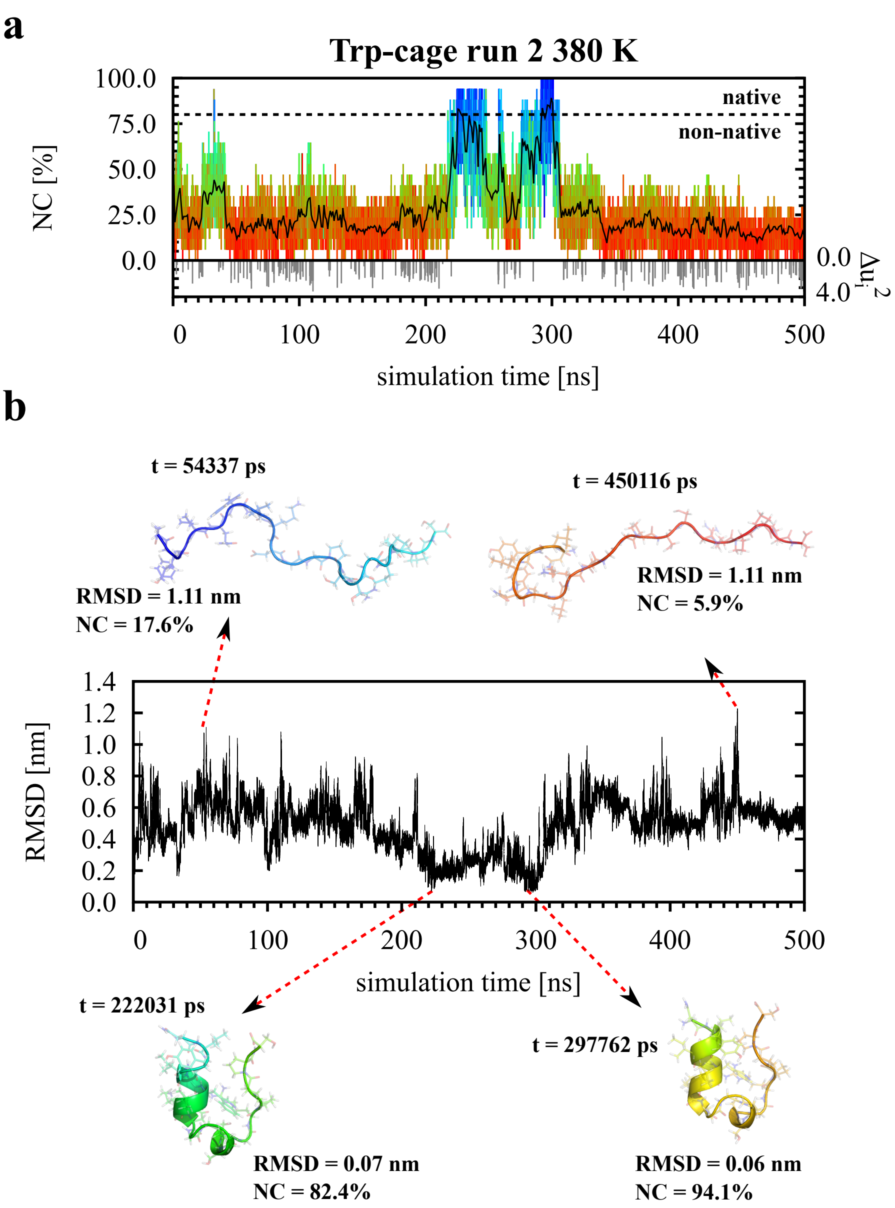

Supplementary Figure 5: (a) Native character (NC) (in %) as a function of simulation time for MD run 2 of the Trp-cage protein at $T = 380$ K. Color line using the BGR palette (from blue, which corresponds to NC = 100%, green, which corresponds to NC = 50%, to red, which corresponds to NC = 0%) represents NC computed every 1 ps and the black line represents the mobile average of the NC computed on a time window of 1 ns. Gray impulses shown on the time axis of the lower graph represent the occurrence of an ILM ($S < 0.5$) along the MD trajectory. (b) Root mean-square deviation ($RMSD$ in nm) as a function of simulation time for MD run 2 of Trp-cage at $T = 380$ K. All-atom structures of Trp-cage for different snapshots are shown in a cartoon plus ball and stick representation. NC for each snapshot is also indicated.

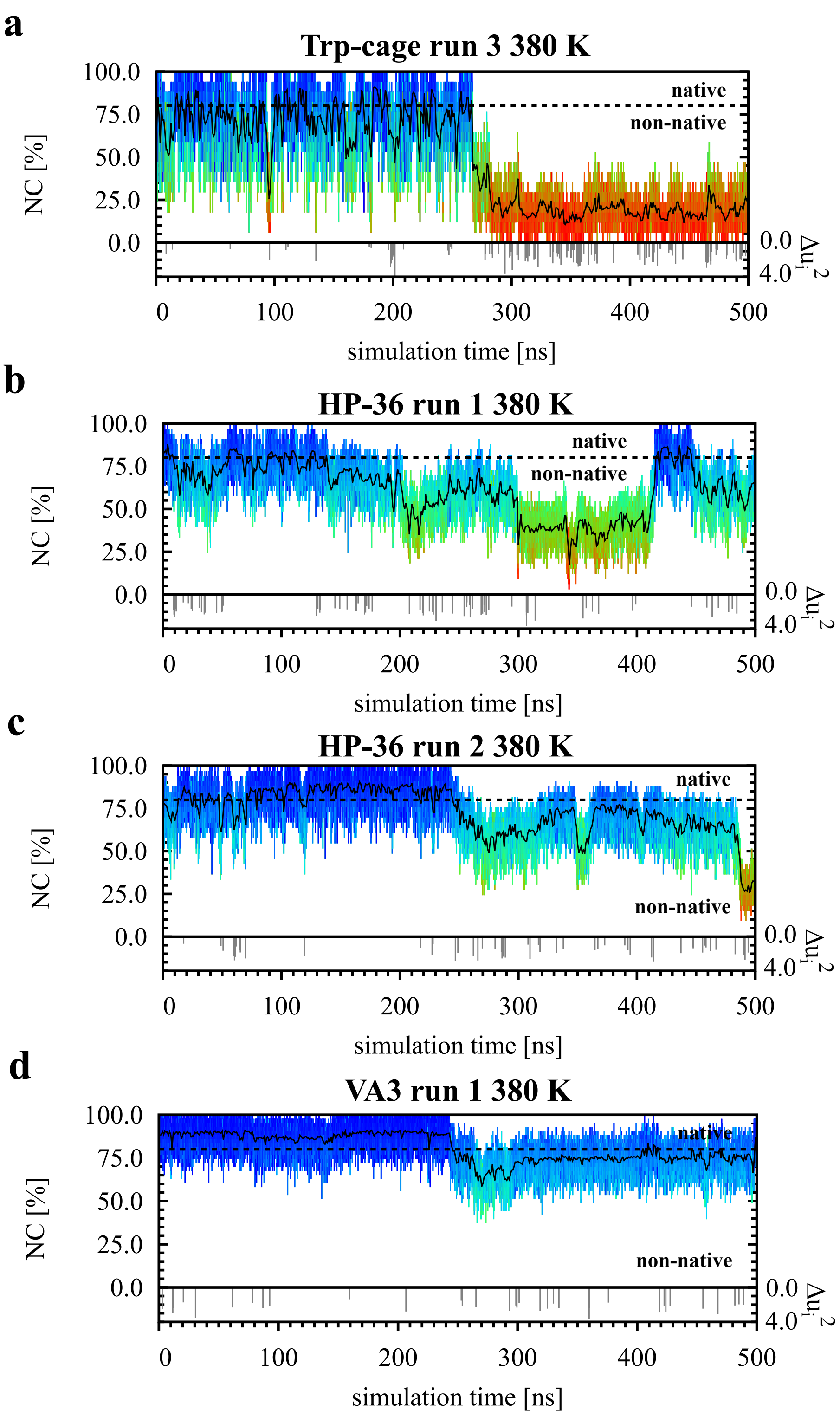

Supplementary Figure 6: Native character (NC) (in %) as a function of simulation time for MD run 3 of Trp-cage (panel a), MD runs 1 and 2 of the HP-36 (panels b and c) and MD run 1 of VA3 (panel d) at T = 380 K. Color line using the BGR palette (from blue, which corresponds to NC = 100%, green, which corresponds to NC = 50%, to red, which corresponds to NC = 0%) represents NC computed every 1 ps and the black line represents the mobile average of the NC computed on a time window of 1 ns. Gray impulses shown on the time axis of the lower graph represent the occurrence of an ILM (S < 0.5) along the MD trajectory.

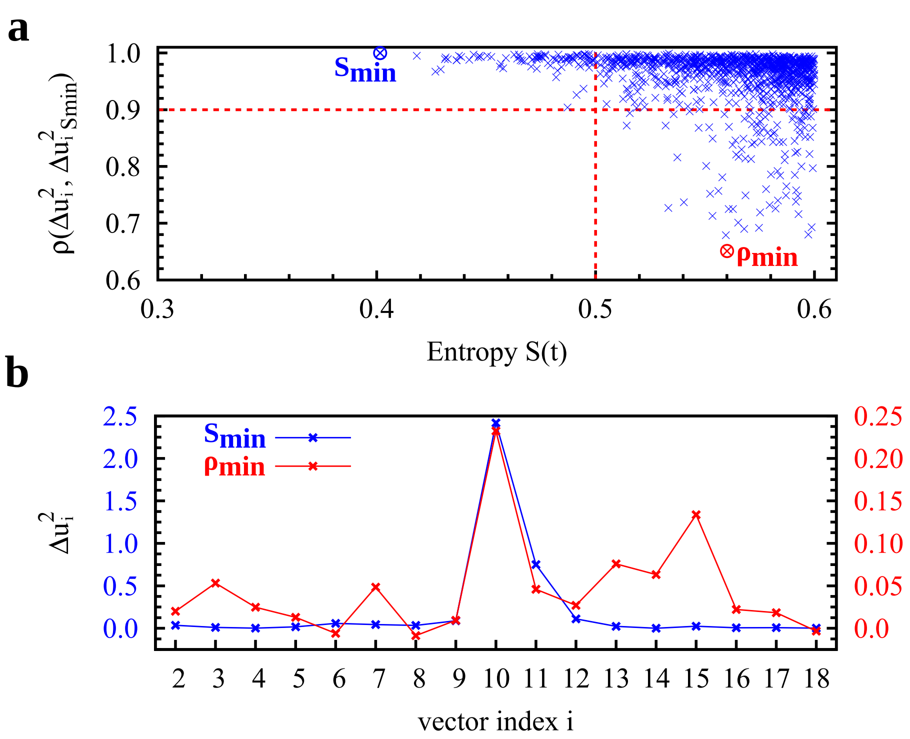

Supplementary Figure 7: Correlation analysis between ILMs. (a) Correlation ρ between $\Delta {u_i}^2$ profiles along the sequence with $S \leq 0.6$ and $\Delta {u_i}^2_{S_{min}}$ profile along the sequence characterized by S minimum ($S = 0.4$). (b) $\Delta {u_i}^2$ profiles along the sequence characterized by S_{min} (blue line) and ρ_{min} (red line), as shown in panel a.

Supplementary Table 1: Number N_{ILM} of ILMs of soliton type detected in MD simulations of the HP-36 protein at $T = 380$ K and their localization along the amino acid sequence (residues involved are listed between parenthesis using the three letters code).

	T = 380 K		T = 300 K
	run 1	run 2	run 1
N_{ILM}	88	65	35
N_{ILM}/N_t (in %)	0.0176	0.0130	0.007
$i = 42$ (MET-LEU-SER-ASP)	1	1	3
$i = 51$ (VAL-PHE-GLY-MET)	3	3	2
$i = 73$ (GLU-LYS-GLY-LEU)	81	59	29
$i = 74$ (LYS-GLY-LEU-PHE)	3	2	1

Supplementary Table 2: Number N_{ILM} of LMs of soliton type detected in MD simulations of VA3 at $T = 380$ K and their localization along the amino acid sequence (residues involved are listed between parenthesis using the three letters code).

	T = 380 K	T = 300 K
	run 1	run 1
N_{ILM}	34	33
N_{ILM}/N_t (in %)	0.0068	0.0066
$i = 19$ (LEU-THR-GLY-ALA)	1	-
$i = 36$ (ILE-SER-GLY-SER)	32	33
$i = 37$ (SER-GLY-SER-THR)	1	-

Supplementary Table 3: Number N_{ILM} of ILMs of the soliton type detected in the MD simulations of Trp-cage and their localization along the amino acid sequence (residues involved are listed between parenthesis using the three letters code). For each mutant, each residue replaced by ALA is marked by a symbol. All MD runs were of a 500 ns duration except run 2, at $T = 300$ K (20 ns, see the Methods section).

	T = 380 K					T = 300 K	
	run 1	run 2	run 3	mutant$^+$	mutant*	run 1	run 2
N_{ILM}	251	302	199	321	58	71	17
N_{ILM}/N_t (in %)	0.0502	0.0602	0.0398	0.0642	0.0116	0.0142	0.0850
$i = 2$ (ASN-LEU-TYR-ILE)	4	3	3	-	6	-	-
$i = 7$ (TRP-LEU-LYS-ASP)	-	1	-	-	-	-	-
$i = 8$ (LEU-LYS-ASP-GLY)	-	-	-	1	-	-	-
$i = 11$ (GLY*-GLY*-PRO-SER)	-	1	-	4	1	-	1
$i = 12$ (GLY*-PRO-SER-SER)	-	1	-	-	1	-	-
$i = 13$ (PRO-SER-SER-GLY*)	-	-	-	1	1	-	-
$i = 15$ (SER-GLY$^{+,*}$-ARG-PRO)	1	2	6	3	-	3	-
$i = 16$ (GLY*-ARG-PRO-PRO)	-	-	1	2	1	-	-
$i = 17$ (ARG-PRO-PRO-PRO)	1	-	1	2	6	4	-

Supplementary Table 4: Number N_{ILM} of ILMs of soliton type detected in MD simulations of the Trp-cage protein using CHARMM27 and AMBER99SB*-ILDN-q force-fields and their localization along the amino acid sequence (residues involved are listed between parenthesis using the three letters code).

	T = 380 K		T = 300 K	
	run CHARMM	run AMBER	run CHARMM	run AMBER
N_{ILM}	254	296	128	74
N_{ILM}/N_t (in %)	0.0508	0.0592	0.0248	0.0148
$i = 2$ (ASN-LEU-TYR-ILE)	3	1	1	-
$i = 7$ (TRP-LEU-LYS-ASP)	-	1	-	-
$i = 9$ (LYS-ASP-GLY-GLY)	18	66	4	-
$i = 10$ (ASP-GLY-GLY-PRO)	57	41	2	-
$i = 11$ (GLY-GLY-PRO-SER)	9	1	5	-
$i = 12$ (GLY-PRO-SER-SER)	-	-	-	-
$i = 13$ (PRO-SER-SER-GLY)	7	2	-	-
$i = 14$ (SER-SER-GLY-ARG)	15	136	-	-
$i = 15$ (SER-GLY-ARG-PRO)	3	3	1	8
$i = 16$ (GLY-ARG-PRO-PRO)	8	-	4	-
$i = 17$ (ARG-PRO-PRO-PRO)	17	5	13	7
$i = 18$ (PRO-PRO-PRO-SER)	117	40	98	59
Solitons $i = 9$,10 and 14	89	243	6	-

Supplementary Table 5: Comparative test of the number of ILMs as a function of the S_{cutoff} value for the run 1 of Trp-cage at 380 K.

S_{cutoff}	N_{ILM}	$i = 9$	$i = 10$	$i = 14$	$i = 18$	N_n	N_{nn}	P_{nn}/P_n
0.45	41	15	16	3	6	-	34	-
0.50	251	95	93	33	24	1	222	27.8
0.55	995	355	376	105	94	12	871	9.3
0.60	3177	997	1138	345	314	77	2736	4.6

Supplementary Movie 1: Typical time evolution of an ILM of soliton type in MD trajectories of Trp-cage of 1 ns duration for which the coordinates were recorded every fs. The time $t = 0$ corresponds to the detection of soliton 3 in the MD trajectory shown in Figure 5 (static dashed line in the video). The variation of Δu_i^2 as function of time between $t = -1$ ps and $t = 2.5$ ps is represented every fs by crosses along the amino acid sequence. The crosses are joined by full lines to emphasize the soliton profile. The time window is identical to the one shown in Figure 6 b and Figure 6 c.
